# HCV Core Protein Uses Multiple Mechanisms to Induce Oxidative Stress in Human Hepatoma Huh7 Cells

**DOI:** 10.3390/v7062745

**Published:** 2015-05-29

**Authors:** Alexander V. Ivanov, Olga A. Smirnova, Irina Y. Petrushanko, Olga N. Ivanova, Inna L. Karpenko, Ekaterina Alekseeva, Irina Sominskaya, Alexander A. Makarov, Birke Bartosch, Sergey N. Kochetkov, Maria G. Isaguliants

**Affiliations:** 1 Engelhardt Institute of Molecular Biology, Russian Academy of Sciences, Vavilov str. 32, Moscow 119991, Russia; E-Mails: o.smirnova.imb@gmail.com (O.A.S.); irina-pva@mail.ru (I.Y.P.); olgaum@yandex.ru (O.N.I.); ilkzkil@gmail.com (I.L.K.); aamakarov@eimb.ru (A.A.M.); kochet@eimb.ru (S.N.K.); 2 Latvian Biomedical Research and Study Center, Ratsupites 1, Riga LV1067, Latvia; E-Mails: kate@biomed.lu.lv (E.A.); irina@biomed.lu.lv (I.S.); 3 Inserm U1052, Cancer Research Center of Lyon, University of Lyon, 151, Cours A Thomas, 69424 Lyon Cedex, Lyon, France; E-Mail: birke.bartosch@inserm.fr; 4 DevWeCan Laboratories of Excellence Network (Labex), Lyon F-69000, France; 5 D.I. Ivanovsky Institute of Virology, Gamaleya str. 16, Moscow 123098, Russia; E-Mail: maria.issagouliantis@ki.se; 6 A. Kirchenstein Institute of Microbiology and Virology, Riga Stradins University, Ratsupites 5, Riga LV-1069, Latvia; 7 Department of Microbiology, Tumor and Cell Biology, Karolinska Institutet, Nobelsvägen 16, Stockholm 17177, Sweden

**Keywords:** hepatitis C virus, oxidative stress, reactive oxygen species, NADPH oxidase, transforming growth factor, cytochrome P450, ER oxidoreductin

## Abstract

Hepatitis C virus (HCV) infection is accompanied by the induction of oxidative stress, mediated by several virus proteins, the most prominent being the nucleocapsid protein (HCV core). Here, using the truncated forms of HCV core, we have delineated several mechanisms by which it induces the oxidative stress. The N-terminal 36 amino acids of HCV core induced TGFβ1-dependent expression of nicotinamide adenine dinucleotide phosphate (NADPH) oxidases 1 and 4, both of which independently contributed to the production of reactive oxygen species (ROS). The same fragment also induced the expression of cyclo-oxygenase 2, which, however, made no input into ROS production. Amino acids 37–191 of HCV core up-regulated the transcription of a ROS generating enzyme cytochrome P450 2E1. Furthermore, the same fragment induced the expression of endoplasmic reticulum oxidoreductin 1α. The latter triggered efflux of Ca2+ from ER to mitochondria via mitochondrial Ca2+ uniporter, leading to generation of superoxide anions, and possibly also H2O2. Suppression of any of these pathways in cells expressing the full-length core protein led to a partial inhibition of ROS production. Thus, HCV core causes oxidative stress via several independent pathways, each mediated by a distinct region of the protein.

## 1. Introduction

Chronic infection with hepatitis C virus (HCV) is characterized by liver fibrosis and cirrhosis, metabolic disorders (steatosis, insulin resistance, iron overload) and liver cancer [1,2,3,4]. Researchers have assigned these pathologies to several HCV proteins including the core protein, which can trigger most of these events individually. HCV core induces cell transformation *in vitro* [5] and liver carcinogenesis in transgenic animals in the absence of inflammation [6]. It is also capable of inducing production of a profibrogenic cytokine-transforming growth factor β1 (TGFβ1), thus leading to activation of hepatic stellate cells (HSCs) and formation of scar tissue in the liver (for example, see [7]). HCV core was shown to transactivate sterol regulatory element binding proteins (SREBP) [8] leading to activated *de novo* synthesis of free fatty acids, and to suppresses peroxisome proliferators-activated receptor (PPAR)-α resulting in impaired fatty acid degradation [3]. This protein is also implicated in blocking expression of a liver hormone hepcidin thus leading to liver iron overload [9]. Therefore, investigation of molecular mechanisms which link HCV core to HCV-induced pathologies is an important goal.

One of the key mechanisms triggering metabolic dysregulation, fibro- and carcinogenesis in HCV infected cells is a virus-induced oxidative stress [1,4,10,11]. Oxidative stress is characterized by the enhanced cellular formation of reactive oxygen species (ROS), which comprise a vast array of molecules and radicals such as hydrogen peroxide (H_2_O_2_), superoxide anion (O_2_•-) and hydroxyl radical (HO•) [12]. These types of ROS are converted into each other by various chemical and enzymatic reactions. Markers of oxidative stress are observed *in vivo* in chronic hepatitis C patients and transgenic mice as well as *in vitro* in cell lines infected with HCV (reviewed in [4,10,11,13]). Levels of oxidative stress markers in liver and serum of the patients correlate with histological activity of the disease. Several viral proteins were shown to affect ROS levels in cells. They include core, NS5A, NS3, E1, E2, and NS4B [4,14,15,16]. However, the major activator of ROS production is HCV core protein (HCV core) [15]. HCV core-induced oxidative stress has been shown to accompany hepatocarcinogenesis [6] and impaired free fatty acid degradation in transgenic mice [11]. Enhanced ROS production in core-expressing cells is crucial for SREBR-mediated cholesterol/sterol biosynthesis as well as for hepcidin down-regulation [9]. HCV core-induced oxidative stress was also shown to induce RNA damage, leading to enhanced HCV genome heterogeneity and allowing the virus to escape immune system and antivirals [17]. However, still little is known about cellular sources of ROS in HCV-infected cells and ROS-induced downstream cascades.

The major sources of ROS in eukaryotic cells include the electron transport chain/oxidative phosphorylation in mitochondria, but also nicotinamide adenine dinucleotide phosphate (NADPH) oxidases (NOX), metabolic enzymes (including xanthine oxidase and enzymes involved in the degradation of lipids and biogenic polyamines), and the folding machinery of endoplasmic reticulum (ER) [12,18,19]. Several of these ROS sources have been implicated in the induction of oxidative stress by HCV. It has been shown that several HCV proteins cause mitochondrial dysfunction [20], induction of NOX1 and NOX4 [21,22], and ER stress [23]. The core protein is localized on the membranes of mitochondria and the ER, on the surface of the lipid droplets and in the nucleus [24,25,26]. Its expression in various human cell lines or direct incubation of core proteins with isolated mitochondria increases ROS production by altering mitochondrial electron transport [16,20] and increases influx of calcium ions [16] by activating the Ca^2+^ uniporter [27] and enhancing efflux of Ca^2+^ ions from ER stores via the induction of ER stress and inhibition of sarco/endoplasmic reticulum Ca^2+^ ATPase 2 [23]. However, the respective importance of these different ROS sources and ROS activating pathways has not been evaluated so far, albeit their importance in disease progression in chronic hepatitis C. So far, most reports concentrated on either mitochondrial or NADPH sources of ROS, whereas additional not yet identified sources of ROS may be activated by HCV.

The major goal of this study was to identify additional sources of ROS, activated by the HCV core, especially outside the mitochondria. A second goal was to identify regions of HCV core responsible for activation of these ROS sources. To achieve this, we designed truncated forms of HCV core protein and tested their effect(s) on the set of regulatory pathways involved in the induction of oxidative stress. We found that in cells expressing the full-length core protein, the key sources of ROS were NADPH oxidases, cytochrome P450 2E1 (CYP2E1), and ER oxidoreductin 1α (Ero1α). Activation of the respective pathways was mediated independently by HCV core fragments encompassing amino acids 1–36 and 37–191.

## 2. Materials and Methods

### 2.1. Materials

Fetal bovine serum (FBS) was obtained from HyClone (Logan, UT, USA). 2′,7′-Dichlorodihydrofluorescein diacetate (DCFHDA) and dihydroethidium (DHE) Sigma (St. Louis, MO, USA), unless otherwise noted. Lipofectamine 2000, MitoSox Red and BAPTA-AM were from Life Technologies (Carlsbad, CA, USA), Ru360 was from Merck-Millipore (Darmstadt, Germany). Primary antibodies to cytochrome P450 2E1 (ab28146), ER oxidoreductin 1α (ab129241), and β-actin (ab3280), anti-rabbit and anti-mouse secondary antibodies conjugated to horseradish peroxidase (HRP), as well as CYP2E1 inhibitor 4-methylpyrazole, were obtained from Abcam (Cambridge, UK). Antibodies to NOX1 (Mox1, H75, sc-25545), NOX4 (H-300, sc-30141), cyclooxygenase 2 (COX-2) (29, sc-19994), and transforming growth factor β1/2 (TGFβ1/2) (12Y-1, sc-80346L) were purchased from Santa-Cruz Biotechnology (Heidelberg, Germany). Immune detection of HCV core was performed using specific antisera raised by immunization of rabbits with the recombinant protein representing aa 1-151 of HCV core [28]. qPCRmix-HS and qPCRmix-HS SYBR+ROX master mixes were from Evrogen (Moscow, Russia).

Huh7 cells were a kind gift from Prof. R. Bartenschlager (Heidelberg University, Heidelberg, Germany).

### 2.2. Plasmids

The plasmid expressing the full-length HCV core protein (pVax1-core(1–191)), corresponding to 1b genotype (GenBank: AF176573), was described previously [29]. To generate the constructs encoding aa 1–36 and aa 37–191 truncated forms of HCV core, the respective fragments were amplified from the plasmid pVax1-core(1–191) using oligonucleotides core1-For (5′-TTTGGATCCGATATGAGCACGAATCCTAAACC-3′), core36-Rev (5′-TTTGAATTCTTACAGGTAAACTCCACCGAC-3′), core37-For (5′-TTTGGATCCGATATGTTGCCGCGCAGGGGC-3′), and core191-Rev (5′-TTTGAATTCTTAAGCGGAAGCTGGGATG-3′). The PCR products were cloned into *BamH*I and *EcoR*I sites of pVax1 vector (Invitrogen, Waltham, MA, USA). The structure of all plasmids was confirmed by sequencing.

### 2.3. Cell Culture and Transfection

Human hepatoma Huh7 cells were maintained and transfected as previously described [15]. For transfection, mixtures of 1 μg of the plasmid with 2 μL of Lipofectamine 2000 were used per well of a 6-well plate. In the case of 24-well plates, a mixture of 0.4 μg plasmid and 0.4 μL Lipofectamine 2000 per well was used. In some experiments, 4-methylpyrazole, BAPTA-AM or Ru360 was added 18 h posttransfection to a final concentration of 100 µM.

### 2.4. Immunoblot Analysis

Western blot analysis was performed as described previously [15]. The primary antibodies were used in the following concentrations: anti-β-actin (0.2 μg/mL), anti-COX-2 (0.4 μg/mL), anti-Ero1α (0.5 μg/mL), anti-NOX1 (0.2 μg/mL), anti-NOX4 (0.4 μg/mL). The HRP-conjugated anti-mouse and anti-rabbit secondary antibodies were used at concentrations of 0.2 μg/mL.

### 2.5. Confocal Microscopy

Transfected cells were grown on glass coverslips. Two days after transfection, the cells were fixed for 20 min at room temperature with PBS containing 4% paraformaldehyde. Fixed cells were permeabilized for 3 min at room temperature with 0.2% Triton X-100 in PBS, followed by blocking with PBS containing 2% bovine serum albumin and 0.2% Tween-20. Permeabilized cells were incubated with anti-core rabbit serum (40 μg/mL) for 60 min at room temperature, followed by incubation for 45 min with fluorescein isothiocyanate (FITC)- or tetramethylrhodamine isothiocyanate (TRITC)-conjugated goat anti-rabbit immunoglobulin G (IgG) (Sigma F9887 and T6778, respectively). Nuclei were visualized by Hoechst 33258 staining. All specimens were examined on a Leica TCS5 laser scanning confocal microscope (Leica, Wetzlar, Germany).

### 2.6. Measurement of Reactive Oxygen Species

Nonspecific measurement of ROS was performed by epifluorescence, using dichlorodihydrofluorescein diacetate (DCFHDA). Huh7 cells were transfected with plasmids expressing HCV core variants. Growth medium was removed 28 h post transfection, removed and cells were incubated at room temperature for 30 min in a fresh medium containing 25 μM DCFHDA, and then washed 10 times with 0.5 mL PBS, and resuspended in 200 μL PBS. The fluorescence intensities (FLI) were recorded on a Plate CHAMELEON V reader (Hidex Ltd., Turku, Finland) with excitation at 485 nm and emission at 535 nm. Specific levels of superoxide anion were measured after treating cells with 25 μM dihydroethidium (DHE) using the protocol given above for DCFHDA. In case of DHE, FLI were measured with excitation at 510 nm and emission at 590 nm. DHE specificity was confirmed by the standard HPLC analysis [30]. Production of superoxide anion in mitochondria was accessed by staining cells with 2 μM MitoSox Red for 30 min with the subsequent fluorescence measurement done as described for DHE. Intracellular levels of H_2_O_2_ in cytoplasm and mitochondria were measured using the genetically encoded fluorescent sensors HyPer-Cyto and HyPer-dMito (Evrogen, Moscow, Russia) by flow cytometry or on the Chameleon V microplate reader. In brief, Huh7 cells seeded on 6-well plates were transfected with a mixture of HCV-core and HyPer-encoding plasmids; 40 h posttransfection, cells were harvested by trypsin, washed with PBS and resuspended in DMEM containing 5% FBS. The level of the oxidized HyPer in the cell was monitored on a Beckman Coulter Epix XL4 flow cytometer (Beckman-Coulter, Brea, CA, USA) as for the eGFP fluorescence (Ex/Em 488/535 nm) [31]. Alternatively, cells were transfected on the 24-well plates as described above, and after 40 h harvested, resuspended in warm PBS and subjected to analysis. The control samples were treated with 100 μM H_2_O_2_ or 250 μM N-acetylcysteine (NAC). Fluorescence was measured in each well at 535 nm after excitation at 485 ± 40 nm and 416 ± 10 nm. HyPer intensities were presented as a ratio of fluorescence at 485 to excitation at 416 nm.

### 2.7. Quantitative RT-PCR (RT-qPCR)

Purification of the total RNA and reverse transcription was carried out as described previously [15]. The resulting cDNA was subjected to quantitative PCR (qPCR) on an iQ5 Real-Time PCR Detection System (Bio-Rad, Hercules, CA, USA). Human NOX1, NOX4, COX-2, TGFβ1, CYP2E1, and Ero1α were quantified by SYBR Green approach, using qPCRmix-HS SYBR+ROX mixture, primer sequences are listed in Table 1. Relative quantitative analysis was carried out by comparing threshold cycle number for target genes and a reference β-actin mRNA, amplified in separate tubes using primers and Taqman probes listed in Table 1. A standard reaction mixture (50 μL) contained the respective primers, the Taqman probe (when required), cDNA equivalent to 100 ng total RNA, and qPCRmix-HS SYBR+ROX or qPCRmix-HS master mix (Taqman protocol). The real-time PCR thermal conditions were 55 °C for 5 min, 95 °C for 10 min, followed by 40 cycles each at 95 °C for 10 s and 57 °C for 1 min (signal collection temperature).

**Table 1:** Primers used for real-time PCR analysis.

Gene Name	Primer/Probe	Sequence ^a^
NOX1	Forward primer	5′-TTAACAGCACGCTGATCCTG-3′
	Reverse primer	5′-CTGGAGAGAATGGAGGCAAG-3′
NOX4	Forward primer	5′-GCTGACGTTGCATGTTTCAG-3′
	Reverse primer	5′-CGGGAGGGTGGGTATCTAA-3′
COX-2	Forward primer	5′-CCATGTCAAAACCGAGGTGTAT-3′
	Reverse primer	5′-TCCGGTGTTGAGCAGTTTTCT-3′
TGFβ	Forward primer	5′-GCAGCACGTGGAGCTGTA-3′
	Reverse primer	5′-CAGCCGGTTGCTGAGGTA-3′
Ero1α	Forward primer	5′-GCATTGAAGAAGGTGAGCAA-3′
	Reverse primer	5′-ATCATGCTTGGTCCACTGAA-3′
CYP2E1	Forward primer	5′-GACTGTGGCCGACCTGTT-3′
	Reverse primer	5′-ACTACGACTGTGCCCTTGG-3′
β-actin	Forward primer	5′-GATCATTGCTCCTCCTGAGC-3′
	Reverse primer	5′-ACTCCTGCTTGCTGATCCAC-3′
	Probe	5′-[R6G]-CTCGCTGTCCACCTTCCAGCAGAT-[BHQ-1]-3′

^a^ R6G, rhodamine 6G; BHQ-1, black hole quencher-1.

### 2.8. RNA Interference

To suppress expression of NOX1, NOX4, COX-2, and Ero1α, the following previously described pairs of oligonucleotides were used: siMock, 5′-GUAAGACACGACUUAUCGCdTdT-3′ and 5′-GCGAUAAGUCGUGUCUUACdTdT-3′; siNOX1, 5′-UCAUAUCAUUGCACAUCUAdTdT-3′ and 5′-UAGAUGUGCAAUGAUAUGAdTdT-3′; siNOX4, 5′-GCCUCUACAUAUGCAAUAAdTdT-3′ and 5′-UUAUUGCAUAUGUAGAGGCdTdT-3′; siCOX2, 5′-UGAAAGGACUUAUGGGUAAdTdT-3′ and 5′-UUACCCAUAAGUCCUUUCAdTdT-3′; siEro1α, 5′-CUGUUUUAAGCCACAGACAdTdT-3′ and 5′-TGTCTGTGGCTTAAAACAGdTdT-3′ [32,33]. Each pair of oligonucleotides (5 µM each) was dissolved in an annealing buffer (5 mM Tris-HCl, pH 7.5, 1 mM EDTA), heated at 65 °C for 5 min and then slowly cooled to room temperature and stored at −80 °C. The Huh7 cells were transfected with 100 pmol of each duplex per well of a 24-well or 400 pmol per well of a 6-well plate, using Lipofectamine2000 according to the manufacturer’s specification.

### 2.9. Statistical Analysis

Statistical analysis was performed with BioStat 2009 software (AnalystSoft, Vancouver, BC, Canada). All data are presented as mean ± SD. Differences between two groups were compared using two-tailed unpaired Student’s *t*-test. For comparison between multiple groups, ANOVA followed with a Tukey-Kramer posttest applied. *p* values < 0.05 were considered statistically significant if not stated otherwise.

## 3. Results

### 3.1. Panel of Truncated Forms of HCV Core

**Figure 1:**
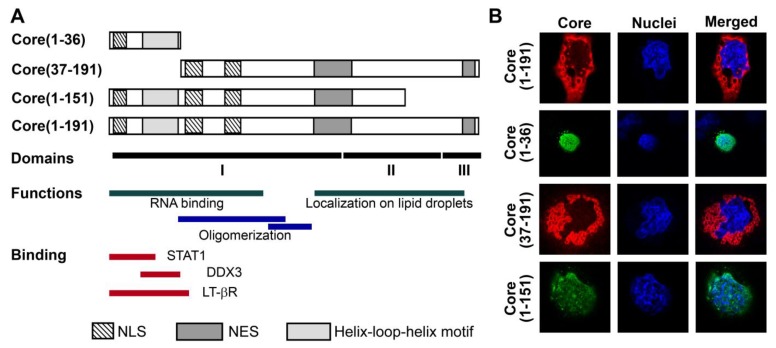
Structure and expression of HCV core variants used to induce oxidative stress and oxidative stress response. (**A**) Schematic representation of HCV core and its truncated forms: the N-terminal fragment aa 1-36, the N-terminally deleted fragment encompassing aa 37–191 and the C-terminally deleted variant encompassing core aa 1–151, dubbed core(1–191), core(1–36), core(37–191) and core(1–151), respectively. Charts below represent the known elements of core structure: structural domains [24], domains responsible for RNA-binding [24], oligomerization [24], localization to lipid droplets [25]; and binding to host proteins STAT1 [36], DDX3 [37], LT-betaR [35]; as well as the position of the helix-loop-helix motif, nuclear localization (NLS) and nuclear export signals (NES) [34]; (**B**) Intracellular localization in Huh7 cells of the full-length and truncated HCV core variants (depicted on the left): staining with primary rabbit anti-core and secondary anti-rabbit antibodies conjugated to FITC or TRITC (panel I), nuclear staining with Hoechst 33258 (panel II), overlay of panels I and II (panel III) (see Materials and Methods Section for details).

We and others have previously shown that expression of HCV core protein in eukaryotic cells induces oxidative stress and activates the Nrf2/ARE antioxidant defense pathway by several, possibly independent, mechanisms [15,16,22]. Mechanistic studies were, however, hampered by simultaneous involvement of HCV core in several ROS-producing and ROS-scavenging processes. To delineate them, we designed a panel of truncated forms of HCV core protein that would activate only a minimum, ideally one, of the pathways. The panel included a peptide representing the N-terminus of the core protein (aa 1–36) with two nuclear localization signals [34] previously implicated in various protein-protein interactions (Figure 1A) [35,36,37]. The other two truncated forms of HCV core were devoid of the N-terminal 36 amino acids as core(37–191), or of the C-terminal 40 amino acids as core(1–151) (note the deletion of ER retention sequence). HCV NS5B served as a negative control, since our previous data demonstrated that this protein has no effect on the production of ROS even expressed to high levels [15].

Expression of the fragments of HCV core in Huh7 cells was confirmed by confocal microscopy using core-specific rabbit antibodies [28] (Figure 1B). Core(1–191) and core(37–191) were localized outside the nucleus, whereas core(1–36) and core(1–151) were predominantly found in the nucleus (Figure 1B). It is also worth noting that a minor fraction of core(1–151) was also located outside nucleus (Figure 1B).

### 3.2. HCV Core Has Two Superoxide-Anion Inducing Domains

**Figure 2:**
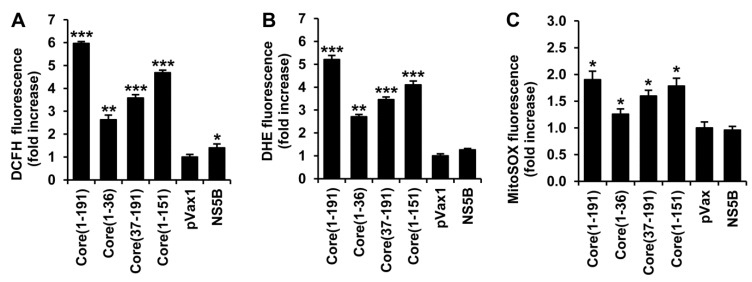

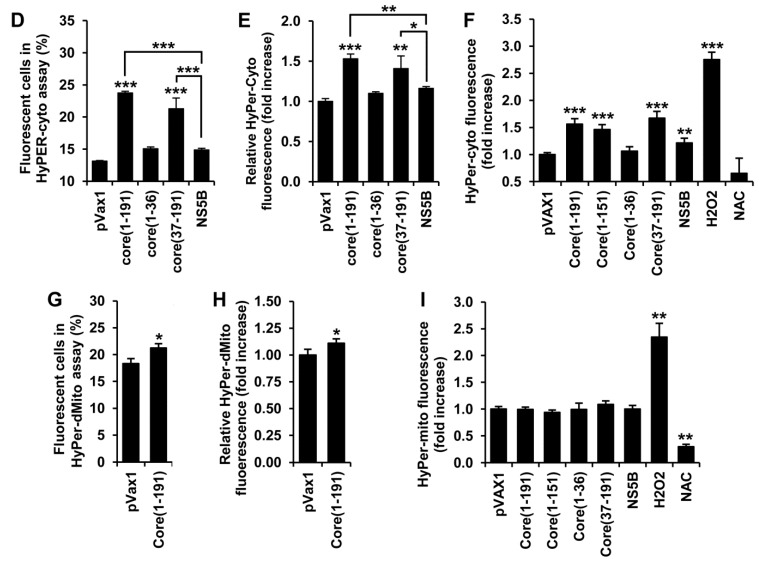
Expression of fragments encompassing HCV core amino acids 1–36 and 37–191 leads to the enhanced production of superoxide anion and hydrogen peroxide. (**A**–**C**) Huh7 cells were seeded on 24-well plates, transfected with plasmids encoding the full-length or truncated forms of HCV core, and 28 h posttransfection stained with one of the dyes: ROS-unspecific 2',7'-dichlorodihydrofluoresceine diacetate (DCFHDA) (**A**), superoxide anion-specific dihydroethidium (DHE) (**B**), or a mitochondrially-targeted superoxide-specific MitoSox Red (**C**), Fluorescence intensity was measured using the Chameleon V microplate reader. Signal was normalized to the levels of fluorescence in Huh7 cells transfected with the empty vector pVax1; (**D**–**I**) Huh7 cells were seeded on 6-well (**D**,**E**,**G**,**H**) or 24-well (**F**,**I**) plates, co-transfected with the plasmid expressing a variant of HCV core and the reporter plasmid expressing a cytoplasmic hydrogen peroxide sensor HyPer-cyto (**D**–**F**) or a mitochondrial hydrogen peroxide sensor HyPer-dMito (**G**–**I**). Percent cells with the elevated level of fluorescence (**D**,**G**), and the relative increase in total fluorescence (**E**,**H**) as compared to untransfected Huh7 cells, were evaluated by flow cytometry. HyPer fluorescence was also measured on a microplate reader at 535 nm after excitation independently at 485 and 416 nm (**F**,**I**). Control samples were treated with H_2_O_2_ or N-acetylcysteine (NAC). Values on each diagram are means ± S.D. of the triplicate measurements done in three independent experiments * *p* < 0.05; ** *p* < 0.01; *** *p* < 0.001.

ROS levels in cells expressing core fragments were quantified using 2',7'-dichlorodihydrofluoresceine diacetate (DCFHDA). DCFHDA penetrates the cells, where it is de-esterified into DCFH; the latter is oxidized by different types of ROS into specific fluorescent products [38]. Both core(1–36) and core(37–191) variants induced a significant elevation of ROS levels (2.6- and 3.6-fold, respectively; Figure 2A), and together added up to the effect of the full-length protein.

Next, we have evaluated the levels of production of one type of ROS, superoxide anion, using a specific dye, dihydroethidium (DHE). Enhanced production of superoxide anion was observed in cells expressing the full-length core(1–191) and all the truncated variants (Figure 2B). Upon interaction with superoxide, DHE forms 2-hydroxyethidium (2-OH-E^+^), but it can also be oxidized to ethidium (E^+^) [38]. HPLC analysis confirmed that the expression of HCV core fragments increased the levels of 2-OH-E^+^ (Supplementary Figure S1 and Table S1). In a similar experiment with MitoSox Red, we measured the production of superoxide anion in the mitochondria. A notable increase in superoxide production was induced by the full-length core(1–191), and the truncated forms core(1–151) and core(37–191) (Figure 2C). A weak but statistically significant increase was also observed for the N-terminal core fragment (Figure 2C).

No ROS production was observed in cells transfected with the empty vector or a control plasmid expressing NS5B (Figure 2A–C). Thus, HCV core appears to have at least two pro-oxidative domains, both capable of inducing superoxide anion, one within aa 37–191 and the other within the N-terminal residues 1 to 36.

### 3.3. HCV Core Fragment aa 37-191 Induces a Strong Production of Hydrogen Peroxide

The impact of various forms of HCV core on the production of H_2_O_2_ was monitored using the genetically encoded fluorescent sensors HyPer-Cyto and HyPer-dMito, expressed in the cytoplasm and in mitochondria, respectively [39]. Huh7 cells were co-transfected with plasmids encoding a variant of core and a HyPer sensor, and 28 h later subjected to analysis by flow cytometry. Co-transfection of the plasmid encoding HyPer-Cyto with the empty vector revealed that approximately 13% of the cells had elevated levels of fluorescence compared to the untreated Huh7 cells (Figure 2D, Supplementary Figure S2A). Co-expression of HyPer-Cyto with the full-length core(1–191) or core(37–191) led to a notable increase both in the number of cells with an elevated level of fluorescence (Figure 2D, Supplementary Figure S2B) and in the mean fluorescence level of the whole cell population (Figure 2E). Only a small, insignificant increase in the levels of fluorescence was observed in Huh7 cells co-expressing HyPer-cyto and core(1–36), or HCV NS5B (Figure 2E). These results point to a significant in-put into ROS production of H_2_O_2_ induced by aa 37 to 191 of HCV core. H_2_O_2_ production by core(37–191) was corroborated by the data on the induction of HyPer-cyto fluorescence obtained on the microplate fluorescence reader (Figure 2F). At the same time, only a very weak increase in fluorescence, if any, was observed in Huh7 cells co-expressing HCV core and HyPer-dMito localized to the mitochondria (Figure 2G–I). Overall, these data demonstrate that the expression of HCV core in Huh7 cells induces the production of H_2_O_2_; the production is triggered by aa 37–191 of the core protein and occurs in the cytoplasm of the expressing cells.

### 3.4. HCV Core Fragment aa 1–36 Mediates the Production of Superoxide Anion by Inducing the Expression of NOX1, NOX4, and TGFβ1

**Figure 3:**
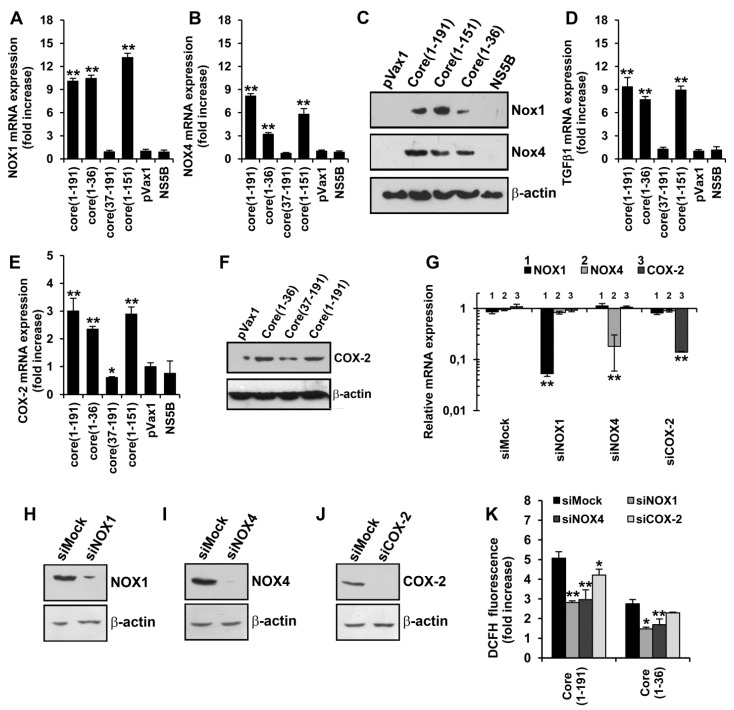

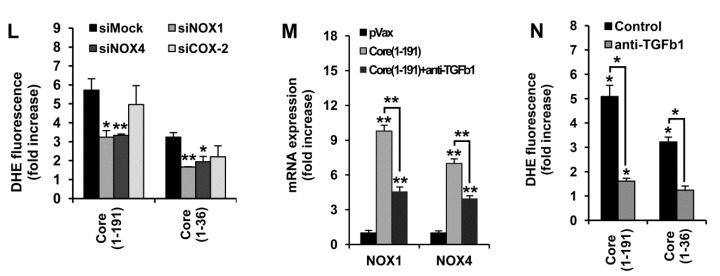
The N-terminus of HCV core protein triggers oxidative stress by independently inducing the expression of NOX1 and NOX4. (**A**–**E**) Quantification in Huh7 cells expressing the full length core(1–191) or truncated forms of HCV core protein core(1–36), core(37–191) or core(1–151) of the mRNA levels of NOX1 (**A**), NOX4 (**B**), TGFβ1 (**D**), and COX-2 (**E**); (**C**,**F**) Up-regulation of the translation of NOX1, NOX4 (**C**), and COX-2 (**F**) in Huh7 cells expressing the full length core(1–191) or truncated forms of HCV core protein core(1–36), core(37–191) or core(1–151) revealed by SDS-PAGE of the respective cell lysates with subsequent Western blotting using antibodies specific to NOX1, NOX4, or COX-2 (as depicted to the right); (**G**) Absence of co-regulation of expression of NOX1, NOX4, and COX-2 demonstrated by a selective inhibition of their expression in Huh7 cells expressing core(1–191) by the specific siRNA (given at the top); (**H**–**J**) siRNAs ensured an effective suppression of expression of the respective protein as revealed by Western blot analysis; (**K**,**L**) DCFH (**K**) and DHE (**L**) staining reveals a partial inhibition of ROS production induced by core(1–191) and core(1–36) in Huh7 cells after silencing of expression of NOX1, or NOX4, or COX-2 with specific siRNAs; (**M**,**N**) Suppression of the expression of NOX1 and NOX4 (**M**) and of superoxide anion production (**N**) in Huh7 cells expressing core(1–191) by antibodies specific to TGFβ1 (see Materials and Methods section for experimental details). Huh7 cells transfected with the empty pVax1 vector or pVax1 expressing NS5B served as negative controls. Levels of NOX1, NOX4, TGFβ1, or COX-2 mRNA were first expressed as relative to the content in the sample of mRNA of β-actin, and then normalized to the relative content of the respective mRNA in Huh7 cells transfected with the empty vector pVax1. Levels of superoxde anion were measured using DHE. All data represent the means ± S.D. from triplicate measurements done in three independent experiments. * *p* < 0.01; ** *p* < 0.001.

Our next step was to identify the molecular mechanisms by which HCV core induces the production of superoxide anion. In the cytoplasm, superoxide anion is generated by various enzymes, including the NOX family of NADPH oxidases. Crucial sources of superoxide anion in HCV-infected hepatocytes are NADPH oxidases 1 and 4 [21,22]. With this in mind, we assessed the effects of HCV core variants on the level of expression of NOX1 and NOX4 using RT-qPCR and Western blot analysis. A significant increase in both NOX1 and NOX4 mRNA and protein levels was observed in Huh7 cells expressing the full-length core(1–191) protein as well as core variants core(1–151) and core(1–36) (Figure 3, panels A–C). No up-regulation was observed in cells expressing core(37–191) or NS5B control (Figure 3A–C).

We also assessed the levels of expression of transforming growth factor β1 (TGFβ1) and cyclo-oxygenase 2 (COX-2), shown to be involved in the regulation of expression of NOX1 and NOX4 [33]. RT-qPCR and Western blotting analysis revealed the transcriptional activation of both genes by core(1–191), core(1–151) and core(1–36) (Figure 3D–F). Thus, we found that HCV core, specifically its N-terminus, activates the expression of the two known cytoplasmic sources of superoxide anion in hepatocytes, namely NOX1 and NOX4, and also of TGFβ1 and COX-2 probably involved in their regulation.

We have further examined the interrelation between HCV core-induced expression of COX-2 and NOX enzymes by down-regulation of the levels of their transcripts using specific siRNAs. Transfection of Huh7 cells with each of the specific siRNA caused a significant reduction of expression of their target genes, as revealed by both RT-qPCR and Western blot analysis (Supplementary Figure S3A–D). It is noteworthy that down-regulation of the expression of any one of them had no effect on the expression of the others (Supplementary Figure S3D). Next, we silenced NOX1, or NOX4, or COX-2 in Huh7 cells expressing HCV core protein. RT-qPCR of Huh7 cells expressing the full-length core protein and super-transfected with siRNAs specific to NOX1, NOX4, or COX-2 demonstrated efficient and specific down-regulation of the corresponding transcripts but not of the others (Figure 3G). This demonstrated that the expression of NOX1 and NOX4 in Huh7 cells expressing HCV core protein does not depend on COX-2.

To specify contribution(s) of NOX1 and NOX4 to the production of ROS, we quantified ROS in Huh7 cells transfected with core variants and super-transfected with siRNA against NOX1 and NOX4 by the total ROS assay with DCFH and by the superoxide-specific DHE assay. Silencing of NOX1 or NOX4 in core(1–191) or core(1–36) expressing Huh7 cells led to a significant 1.6-1.9 fold suppression of the production of total ROS and of superoxide anion (Figure 3H,I). Silencing of COX-2, shown to have no effect on the expression of either NOX1 or NOX4 (Figure 3G), had no effect on the production of ROS (the inhibitory effect of silencing on ROS production never exceeded 20%; Figure 3H,I). Thus, the N-terminal fragment of HCV core induced NOX1- and NOX4-mediated COX-2 independent production of superoxide anion.

Previously, it was shown that TGFβ plays a crucial role in the induction of NOX4 [22]. We have examined the interrelation of expression of TGFβ1 and of NOX1 and NOX4 in the presence of HCV core by treating core(1–191) expressing Huh7 cells with antibodies neutralizing TGFβ1/2. This led to an expected decrease in the expression of NOX4, and also of NOX1 (Figure 3J). This indicated that the HCV core induced up-regulation of NOX enzymes could have been mediated by TGFβ1. The effect of neutralization of TGFβ1 on the production of superoxide anion was verified in DHE assay. It relealed that TGFβ1 is indeed involved in NOX1- and NOX4-mediated ROS production in core-expressing cells (Figure 3N).

### 3.5. Core Amino Acids 37-191 Trigger the Production of ROS by Inducing the Expression of Cytochrome P450 2E1 and Ero1α

**Figure 4:**
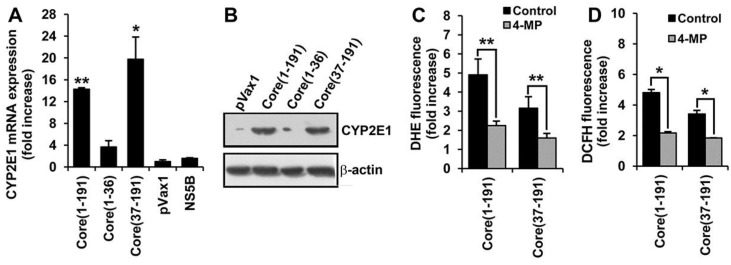
Fragment encompassing aa residues 37–191 of HCV core contributes to ROS production by activating the expression of cytochrome P450 2E1 (CYP2E1). (**A**,**B**) Up-regulation of CYP2E1 expression in Huh7 cells transfected with plasmids expressing core(1–191) and core(37–191) but not core(1–36) or NS5B protein control assessed by the real-time-qPCR (**A**) and Western blotting (**B**); The levels of CYP2E1 mRNA assessed by RT-qPCR (see Materials and Methods) are represented as relative to the expression of β-actin; the resultant values are further normalized to the relative expression of CYP2E1 in Huh7 cells transfected with the empty vector pVax1; (**C**,**D**) CYP2E1 inhibitor 4-methylpyrazole (4-MP) suppressed the production of superoxide anion (**C**) and ROS (**D**) in Huh7 cells expressing the full-length core(1–191) and the N-terminally truncated variant core(37–191). Fluorescence levels are expressed as a fold-increase compared to the mock-treated Huh7 cells transfected with the empty vector pVax1. All data represent the means ± S.D. from the triplicate measurements done in three independent experiments. * *p* < 0.01; ** *p* < 0.001.

We have next examined whether the induction of oxidative stress by HCV core protein could be mediated by ROS-generating enzymes other than NADPH oxidases. One of the candidate inducers of ROS is a cytochrome P450 isoform 2E1 (CYP2E1) [40,41]. Indeed, we found that expression of the full-length core and core(37–191) resulted in a substantial increase in the mRNA and protein levels of CYP2E1 (Figure 4A,B). No significant up-regulation was observed upon the expression of core(1–36) or NS5B control. A two-fold reduction of ROS and superoxide anion levels in core(1–191)- and core(37–191)-expressing cells was observed upon treatment of expressing cells with a specific inhibitor of CYP2E1 activity 4-methylpyrazole (4-MP) [42] (Figure 4C,D). As in the case of NOX1 and NOX4, siRNA silencing of CYP2E1 in HCV core-expressing Huh7 cells also resulted in a decrease in the levels of ROS in the DCFH-, and of the superoxide anion in the DHE-based assay (data not shown). Thus, cytochrome P450 isoform 2E1 triggers the production in Huh7 cells of superoxide anion (and possibly of the other types of ROS).

**Figure 5:**
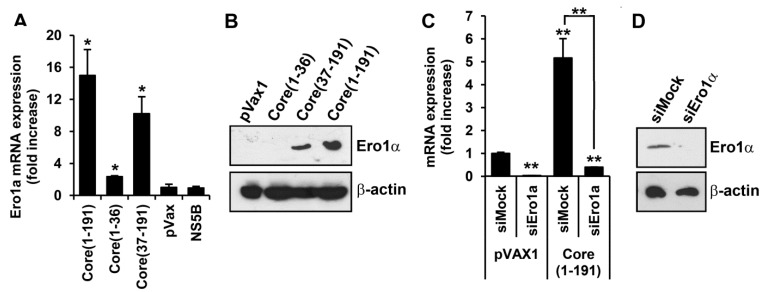

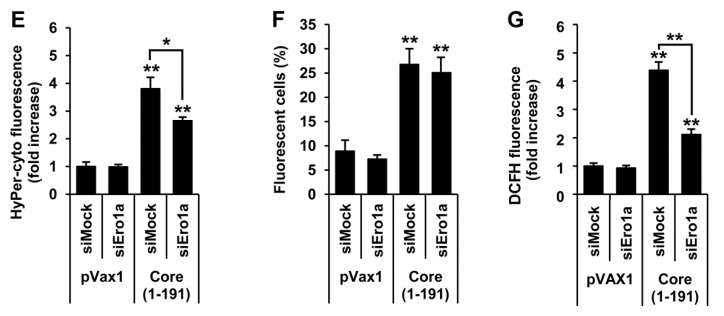
Fragment encompassing aa 37–191 of HCV core is responsible for the activation of ER oxidoreductin 1α (Ero1α) which contributes to the production of hydrogen peroxide. (**A**,**B**) Induction of the expression of Ero1α assessed by RT-qPCR (**A**) and Western-blotting (**B**) of Huh7 cells 40 h post transfection with plasmids expressing HCV core; (**C**,**D**) Silencing of Ero1α expression in naïve and HCV core-expressing Huh7 cells. Respective cells were transfected with anti-Ero1α siRNA or mock siRNA as a negative control; expression of Ero1α was analyzed by RT-qPCR (**C**), and for HCV core expressing cells also by Western-blotting (**D**), values were normalized to the expression of β-actin; (**E**–**G**) Silencing of Ero1α in HCV core expressing Huh7 cells using specific siRNA (siEro1a) inhibits the production of hydrogen peroxide; no effect is observed after treatment of cells with mock siRNA (siMock). To assess this, Huh7 cells were transfected with the respective siRNA and a mixture of plasmids expressing core(1–191) and HyPer-cyto sensor; mean fold increase in HyPer-cyto fluorescence (**E**) and the % of fluorescent cells (**F**) was quantified by flow cytometry. Alternatively, Huh7 cells were transfected with the plasmid expressing core(1–191) and a mixture of respective siRNA; the induction of ROS was assessed by the DCFH assay (**G**). Fluorescence was expressed as a fold-increase compared to the fluorescence measured in mock-treated Huh7 cells transfected with the empty vector pVax1. For experimental details, see Materials and Methods. All data represent the means ± S.D. from triplicate measurements done in three independent experiments. * *p* < 0.01; ** *p* < 0.001.

In an independent set of experiments, we tested whether the N-terminally deleted core could also induce other ROS types, namely hydrogen peroxide. We have demonstrated that the major source of H_2_O_2_ production in cells expressing HCV core is located outside mitochondria (Figure 2). One of the key sources of hydrogen peroxide outside mitochondria is the ER oxidoreductin 1α (Ero1α) [19]. RT-qPCR and Western blot analysis demonstrated that both the full-length and the N-terminally deleted core variants induce the expression of Ero1α (Figure 5A,B). To estimate the impact of Ero1α on the production of ROS, we silenced its expression with siRNA. The level of Ero1α in the “naïve” Huh7 cells transfected with Ero1α-specific siRNA decreased 30 times, and in the HCV core expressing Huh7 cells, 12 times, compared to the respective cells treated with mock siRNA (Figure 5С). Suppression of the expression of Ero1α coincided with a significant reduction of the production of H_2_O_2_, as was registered in the transfection of Ero1α-silenced core-expressing Huh7 cells with a plasmid encoding HyPer-Cyto (Figure 5E). It is noteworthy that treatment with Ero1α-specific siRNA affected only the levels of fluorescence (Figure 5E), not the number of fluorescent cells (Figure 5F). The latter indicated that Ero1α silencing had a specific effect on the ROS-producing/fluorescent cell population. A similar result was obtained in the DCFH assay (Figure 5G). Overall, this demonstrated that the N-terminally deleted core protein can trigger both the production of superoxide anion mediated by CYP2E1 and the production of H_2_O_2_ mediated by Ero1α.

### 3.6. Core aa 37–191 Fragment Triggers the Production of Superoxide Anion by Ero1α-Mediated Efflux of Calcium Ions

**Figure 6:**
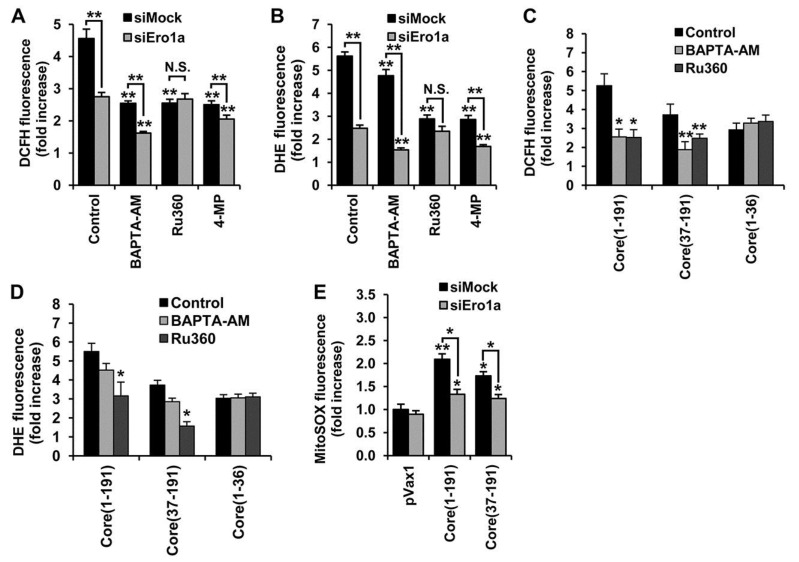
Amino acids 37–191 of the HCV core are responsible for the activation of ER oxidoreductin 1α (Ero1α), redistribution of calcium ions between ER and mitochondria, and the production of superoxide anion and hydrogen peroxide. (**A**,**B**) DCFH (**A**) or DHE assays (**B**) demonstrate that ROS production by core(1–191) expressing Huh7 cells with siRNA-silenced Ero1α expression is partially inhibited by calcium chelator BAPTA-AM and by CYP2E1 inhibitor 4-methylpyrazole (4-MP), but not by the inhibitor of mitochondrial calcium uniporter Ru360; (**C**,**D**) The inhibitory effect of calcium chelator BAPTA-AM on the total ROS production in DCFH (**C**), and superoxide anion in the DHE assay (**D**), is mediated on Huh7 cells expressing core(1–191) and core(37–191), but not core(1–36); (**E**) Suppression of Ero1α expression with siRNA in cells expressing core(1–191) or core(37–191) results in a partial decrease in the production of superoxide anion in mitochondria; results of the MitoSOX assay. Huh7 cells transfected with the empty vector pVax1 or a plasmid encoding HCV NS5B served as negative controls. Fluorescence was expressed as a fold-increase compared to the fluorescence measured in mock-treated Huh7 cells transfected with the empty vector pVax1. For experimental details, see Materials and Methods. All data represent the means ± S.D. from triplicate measurements done in three independent experiments. * *p* < 0.01; ** *p* < 0.001. N.S., not significant.

There is growing evidence that, in a healthy cell, Ero1α regulates the flux of calcium ions from ER to mitochondria [32,43]. An increase in Ca^2+^ levels in mitochondria triggers its dysfunction and constitutes the major, most well studied source of ROS in the HCV-infected (HCV core-expressing) cells [27]. To estimate the impact on ROS production of the HCV-core induced redistribution of calcium ions between the organelles, we used a cell-permeable cytoplasmic calcium ion chelator (BAPTA-AM), and an inhibitor of mitochondrial calcium uniporter (Ru360), assisted by unspecific (siMock) and Ero1α-specific (siEro1α) siRNAs. Treatment of HCV core-expressing mock siRNA super-transfected cells with either of the compounds suppressed the production of ROS as revealed by the DCFH assay (Figure 6A, black bars). The DHE assay demonstrated that treatment of core-expressing cells with Ru360 significantly reduces the production of superoxide anion, whereas only slight inhibition is seen in cells treated with BAPTA-AM (Figure 6B, black bars). Importantly, this was also observed for the BAPTA-AM and Ru360 treated Huh7 cells expressing core(37–191), but not core(1–36) (Figure 6C,D). 

The DCFH and DHE assays revealed that silencing of Ero1α expression by anti-Ero1α siRNA made the production of ROS insensitive to the inhibitor of the mitochondrial calcium uniporter (Ru360; Figure 6A,B). Furthermore, siEro1α treatment of HCV core-expressing Huh7 cells caused a notable decrease of fluorescence in the MitoSox Red assay (Figure 6E), suggesting that anti-Ero1α siRNA mediated a decrease of superoxide levels in mitochondria. On the other hand, silencing of Ero1α had no effect on the inhibition of calcium ion flux by the cytoplasmic calcium ion chelator BAPTA-AM (Figure 6A,B). Taken together, these findings indicate that the production of ROS, specifically of superoxide anion, can be triggered by the full-length HCV core as well as by the N-terminally truncated core(37–191) and depends (at least in part) on the activity of Ero1α, namely on the Ero1α-mediated efflux of calcium ions to mitochondria.

Since HCV core protein induced both Ero1α and CYP2E1, it could not be excluded that these two ROS-generating pathways are interrelated. Possible interaction between Ero1α and CYP2E1 was addressed using a combination of treatments with anti-Ero1α siRNA and CYP2E1 inhibitor 4-methylpyrazole. Treatment with 4-MP suppressed ROS production in the core-expressing Huh7 cells transfected with anti-Ero1α as well as with mock siRNAs (Figure 6A,B), indicating an absence of synergism between these ROS-generating enzymes.

## 4. Discussion

The mechanisms of induction of oxidative stress in HCV infection play a major role in the associated pathology but are not yet fully elucidated. Several studies have shown that oxidative stress in HCV-infected cells is caused by specific viral proteins and, in particular, by HCV core (reviewed in [4]). So far, several sources of ROS have been described in core-expressing cells such as NADPH oxidases [21,22] and the mitochondrial oxidative phosphorylation respiratory complexes [16]. The aim of this study was to identify additional sources of ROS induced by HCV core protein and to decipher the underlying molecular mechanisms.

**Figure 7:**
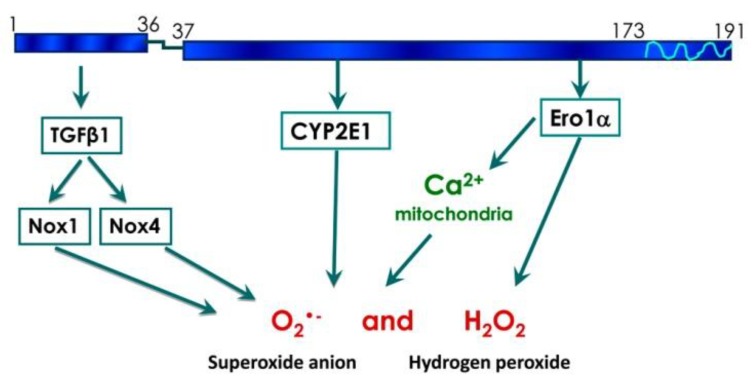
Proposed model of regulation of oxidative stress by HCV core protein.

Two of the sources of ROS in HCV core-expressing cells are NADPH oxidases NOX1 and NOX4 [21,22]. We could assign the NOX-inducing activity of core to its N-terminal fragment, a region of the core protein (Figure 7) that is already implicated in the interaction with a variety of proteins, including DDX3 helicase [37], lymphotoxin receptor [35] and STAT1 [36]. The induction of NOX1 and NOX4 by core(1–36) was accompanied by an increase in the production of O_2_•-, but not of H_2_O_2_. NOX4, in contrast to other NOX enzymes, has been described in the literature to produce H_2_O_2_ due to an extended E-loop 3 of the protein but not to produce O_2_•- [44]. This finding has been challenged by a number of reports that suggest that NOX4 produces O_2_•- (for example, see [45]). In particular, de Monchel *et al.* [21] reported a five-fold decrease in O_2_•- levels in cells with reduced expression of either NOX1 or NOX4. Our findings described here corroborate the fact that NOX4 can produce O_2_•-. However of course, we cannot exclude that NOX4 contributes to O_2_•- production not directly but indirectly via activation of superoxide-producing enzymes. However, a recent paper by Y. Nicimoto *et al.* showed that NOX4 converts an oxygen molecule into hydrogen peroxide by two single-electron transfer steps [46]. In some cases, the intermediate superoxide product is released before transfer of the second electron thus enabling NOX4 to generate both hydrogen peroxide and O_2_•-. Interestingly, I.Helmcke *et al.* described that substitution of the transmembrane domain (TMD) 1 of NOX4 with the TMD1 of NOX1 enables the enzyme to produce O_2_•- as a main product [47], and an alternatively spliced NOX4 form lacking transmembrane domains has been reported to produce O_2_•- [48]. We have also investigated the induction of the TGFβ1→NOX1→COX-2→NOX4 cascade in core-expressing Huh7 cells. This cascade has been shown to be activated in Chang liver cells in response to pro-inflammatory and toxic insults [33], and dependence of NOX4 expression on TGFβ1 has also been shown by other studies [21]. Here, we found that down-regulation of the expression of TGFβ1 affected the expression of both NOX1 and NOX4, confirming the existence of TGFβ1→NOX4 and TGFβ1→NOX1 “subcascades”. Both full-length and the N-terminal (1–36) fragment of HCV core induced the expression of COX-2. However, knock-down of NOX1 and COX-2 using siRNAs had no obvious effects on the suggested “downstream” targets [33]. Furthermore, knock-down of COX-2 did not alter ROS levels, suggesting that the TGFβ1→NOX1→COX-2→NOX4 cascade is not active in HCV-core expressing Huh7 cells. The deviation of our data from that of Chen CL *et al.* [33] could be due to the differences in the source of stress and the cell lines used for the studies.

We identified cytochrome P450 2E1 (CYP2E1) as a novel source of ROS in HCV core expressing cells. This enzyme is localized in the ER and mitochondria and drives the detoxification of various low molecular weight compounds including ethanol, acetone and fatty acids [40,41]. The catabolism of all CYP2E1 substrates leads to the production of O_2_•- and H_2_O_2_ [40,41]. Levels of CYP2E1 mRNA are known to be elevated in biopsies of hepatitis C patients with mild fibrosis [49]. Here, we show that CYP2E1 induction by HCV is due to HCV core protein, and that CYP2E1 is an important source of ROS in infected cells.

Standard sources of H_2_O_2_ are superoxide dismutases (SOD) [13], enzymes of various metabolic pathways (for example, [18]), or the oxidative folding machinery in the endoplasmic reticulum [12,19]. We found that full-length core and core fragments (37–191) induced Ero1α, a component of the folding machinery. Knock-down of Ero1α reduced H_2_O_2_ levels in the cytoplasm. This may either be due to increased production of H_2_O_2_ at the ER and subsequent leakage into the cytoplasm. Alternatively, increased H_2_O_2_ levels in the cytoplasm may indicate the existence of yet unknown cytoplasmic sources of hydrogen peroxide driven by Ero1α. Interestingly, Anelli *et al.* revealed that Ero1α is involved in the control of the direct translocation of calcium ions from ER to mitochondria [32]. An increase in Ca^2+^ levels in mitochondria triggers their dysfunction and is a major source of ROS in HCV-infected and HCV core-expressing cells [27]. Corroborating these data, we observed that the HCV core-induced production of ROS was effectively suppressed by Ru360, which inhibits direct translocation of calcium ions from ER to mitochondria; Ru360 lost its activity when Ero1α expression was suppressed with a specific siRNA. This demonstrated that HCV core may induce oxidative stress by enhancing the Ero1α-mediated efflux of calcium ions into mitochondria, thus inducing mitochondrial dysfunctioning.

We did not observe any notable effect of HCV core on the H_2_O_2_ levels in mitochondria. This may be due to the induction of mitochondrial H_2_O_2_ scavengers. Indeed, HCV and core protein were shown to activate the Nrf2/ARE defense pathway [15,50], which controls the expression of a variety of H_2_O_2_-scavenging enzymes, including several peroxiredoxins such as Prdx1, 3, 5 and 6 and glutathione peroxidases (GPx) 2 and 4 [51,52]. Accordingly, HCV-infected cells exhibit higher levels of at least mitochondrial Prx5 [53] and GPx4 [54].

Extensive data emerging in redox biology field evidence that production of ROS plays an important role in development of various pathologies. According to them, induction of NOX1 and NOX4 shown here might represent a mechanisms of fibrogenesis [55], since these oxidases were previously shown to be crucial for activation of hepatic stellate cells [56]. In addition, NOX4 was shown to be especially critical for TGFβ1-triggered apoptotic death of hepatocytes thus contributing to development of liver disease [56]. An inhibition of NOX1 and NOX4 with newly developed compound GKT137831 was shown to attenuate both hepatocyte death and fibrosis progression *in vivo* [57]. Induction of CYP2E1 by HCV core can play a role in liver disease progression, especially in patients addicted to chronic alcohol consumption (reviewed in [10]). Indeed, co-expression of both proteins leads to augmented oxidative stress [58]. Induction of CYP2E1 in HepG2 cells or in primary murine hepatocytes and concomitant enhanced ROS production were shown to trigger activation of hepatic stellate cells (HSCs) thus contributing to fibrogenesis [59,60]. Indeed, a correlation of CYP2E1 expression in liver biopsies from chronic hepatitis C carriers with liver inflammation and fibrosis score was reported [61], although contradictory data also exist [62]. Similar data about increased CYP2E1 expression in livers during development of steatosis were also reported [63]. Importantly, inhibition of CYP2E1 in hepatocytes with low molecular weight compounds is regarded as a promising strategy for prevention/therapy of liver fibrosis. It was exemplified by prevention of HSC activation with diethylthiocarbamate in HSC-hepatocytes co-cultivation assay [64]. Activation of Ero1α might contribute to enhanced hepatocyte apoptosis most likely due to calcium leakage from the ER, accumulation in mitochondria and subsequent dysfunction of this organelle [43,65]. Increased expression of Ero1α has been also previously shown for breast and various gastrointestinal cancer, with expression levels correlating with tumor growth and metastasis thus being probably involved in HCV-induced carcinogenesis and tumor progression [66,67]. Induction of Ero1α may also be beneficial for the virus since it improves protein folding and secretion, as was exemplified by both production of endogenous proinsulin in pancreatic β-cells [68] and of recombinant heterologous proteins in CHO cell line [69]. Finally, an induction of Ero1α residing at mitochondria-associated membranes (MAM) allows the assumption that it may also affect MAM integrity. E. Tubbs recently showed that such alterations in hepatocytes dysregulate insulin signalling and promotes insulin resistance [70]. If so, this mechanism may contribute to development of a chronic hepatitis C-associated insulin resistance and diabetis mellitus. However, all these speculations require verification in the future.

## Conclusions

Thus, we have shown that HCV core protein triggers ROS production by multiple mechanisms. This fact may constitute a critical element in the life cycle of the virus and be a major trigger of HCV pathophysiology. The role of endogenous ROS and ROS-generating and scavenging enzymes in HCV propagation will therefore remain an important subject of our future studies.

## Supplementary Files

Supplementary File 1
