# Supplementary material for: HCV Core Protein Uses Multiple Mechanisms to Induce Oxidative Stress in Human Hepatoma Huh7 Cells
Source: Viruses. 2015 May 29;7(6):2745–70. doi: 10.3390/v7062745 (PMC4488712; doi:10.3390/v7062745)
Supplement: Supplementary File 1 [file viruses-07-02745-s001.pdf]

SUPPLEMENTARY MATERIAL

Table S1

HPLC analysis of DHE oxidation products in Huh7 cells transfected with pVax1 or a plasmid expressing the full-length HCV core protein

Plasmid, used for transfection of Huh7 cells	Peak Intensity (mV*s)	
	$2\text{-}OH\text{-}E^+$	E^+
pVax1	0.09 ± 0.02 (3)	0.83 ± 0.19 (3)
pCMV-Core(1-191)	0.79 ± 0.18 (3)	0.16 ± 0.06 (3)

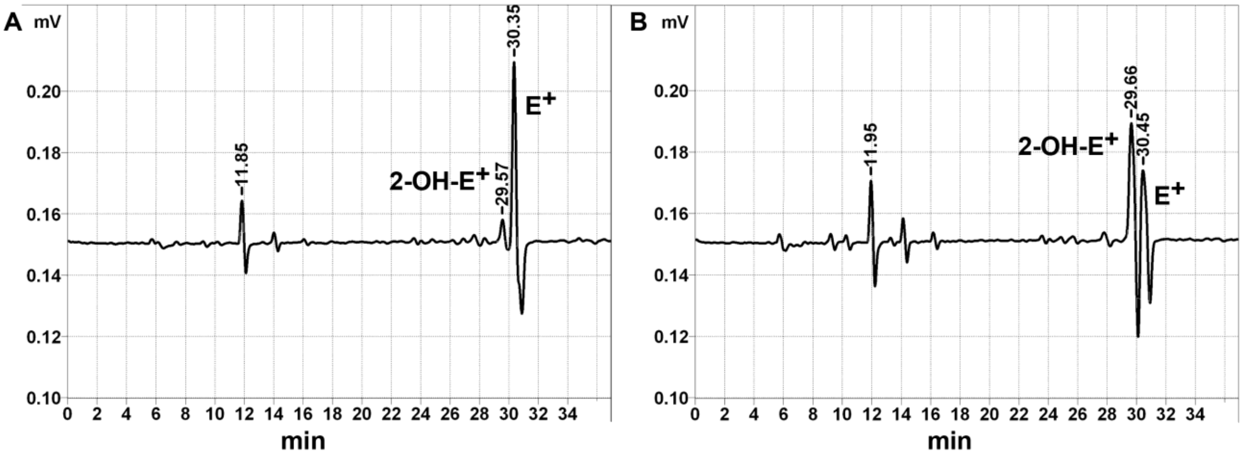

Fig. S1. HPLC chromatogram of the extract of Huh7 cells transfected with pVax1 vector or a plasmid expressing HCV core protein. Huh7 cells were seeded on 100 mm culture dishes, transfected with the plasmid expressing core(1-191) or an empty pVax1 vector, treated 36 h post-transfection with DHE for 10 min and harvested. The samples were prepared and analyzed by HPLC as described by J. Zielonka *et al* (Nature Protocols, 2008). The superoxide-specific ($2\text{-}OH\text{-}E^+$) and unspecific (E^+) products were detected by measurement of fluorescence at 510/595 nm.

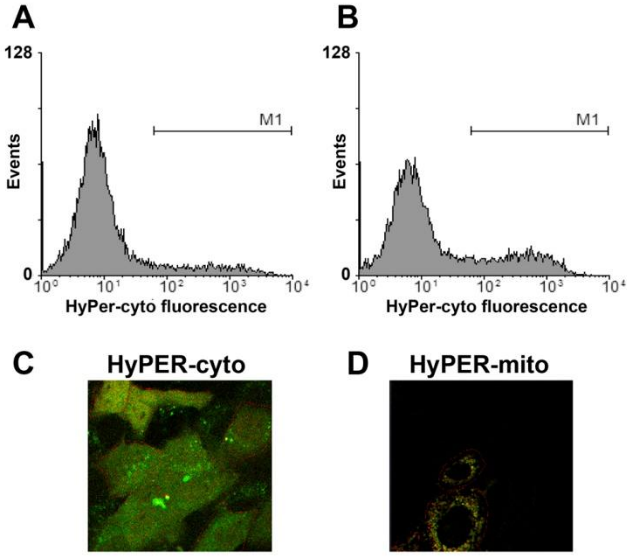

Figure S2. Representative distribution of the fluorescence levels in Huh7 cells co-transfected with HyPer-cyto and pVax1 (**A**) or core(1-191) expressing plasmid (**B**) evaluated by flow cytometry with excitation at 488 nm and detection at 535 nm. Gate M1 was set to exclude >99% of the untransfected Huh7 cells.

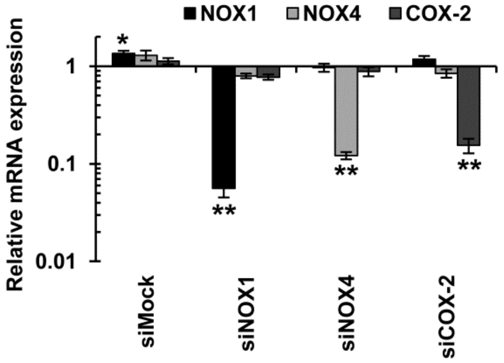

Figure S3. Down-regulation of the expression of NOX1, NOX4, and COX-2 genes using respective siRNAs. Huh7 cells were transfected with siRNA against NOX1, NOX4, or COX-2 siRNAs. Huh7 cells transfected with mock siRNA served as a negative control. Expression of the respective genes was analyzed by RT-qPCR and normalized to the expression of β-actin. The data represent the means ± S.D. from the triplicate measurements done in three independent experiments. **P* < 0.01; ***P* < 0.001.
